# Nutrition literacy profile among adolescents in Kuwait: a cross-sectional study

**DOI:** 10.3389/fpubh.2024.1453484

**Published:** 2024-12-09

**Authors:** Anwar H. AlBaloul, Maraheb M. AlKhalidi, Haya AlAjmi

**Affiliations:** Department of Community Medicine and Behavioral Sciences, Faculty of Medicine, Kuwait University, Safat, Kuwait

**Keywords:** adolescent, nutrition literacy, nutrition knowledge, body mass index, sugar-sweetened beverages

## Abstract

**Background:**

Adolescent nutrition literacy and knowledge are associated with nutrient intake and obesity. With the rising prevalence of obesity in Kuwait, limited studies have assessed nutrition literacy among Kuwaiti adolescents. Therefore, this study aims to assess nutrition literacy among Kuwaiti adolescents and examine factors associated with nutrition literacy, such as body mass index (BMI) and dietary intake.

**Methods:**

A school-based cross-sectional study was conducted in public schools across Kuwait, enrolling a convenience sample of 375 adolescents (mean age = 15.6 years, 53.1% female participants). A self-administered questionnaire was used to collect relevant data. The Nutrition Literacy Assessment Instrument (NLit) score was used to assess nutrition knowledge and skills in making food choices.

**Results:**

The median NLit score among adolescents was 29 (IQR = 6). A large percentage (72.5%) of Kuwaiti adolescents demonstrated adequate overall nutrition literacy and scored adequately in the majority of NLit subscales. However, the majority of adolescents (73.3%) demonstrated inadequate nutrition literacy in understanding food labels. Predictors of NLit included BMI, place of residence, and smoking status. Moreover, the NLit score was negatively associated with the intake of sugar-sweetened beverages.

**Conclusion:**

This study’s findings may inform future research aimed at designing effective nutrition literacy interventions to enhance food choices among adolescents in Kuwait. The results of this study may also provide insights for policymakers and public health professionals to tailor food and nutrition programs to address the decline in food literacy skills and combat obesity.

## 1 Introduction

Adolescence is a transitional phase from childhood to adulthood. During this period, the majority of adolescents experience physiological changes and rapid growth, accompanied by behavioral changes, such as increased independence in decision-making (1). The physiological changes and growth in adolescence create specific nutritional requirements (2). Failing to meet these nutritional requirements has been associated with a higher incidence of several health outcomes (3, 4). Soltani et al. showed that poor dietary choices are associated with higher adolescent central obesity (4), while Barnes et al. showed abnormal blood glucose levels among adolescents with poor dietary habits (3). Adolescent independence in decision-making is a key factor influencing their dietary choice (5). Therefore, equipping adolescents with essential health and nutrition skills to guide their dietary decisions is crucial in preventing adverse health outcomes.

Factors contributing to poor dietary behavior among adolescents are complex and multifactorial, requiring a broader approach to assess its shortfalls. Previous studies proposed that 19% of adolescent food choices are affected by their peers (6, 7). In addition, adolescent diet quality was associated with parents’ nutritional literacy (8, 9). Among those individual factors, nutrition literacy or knowledge has been identified as a potential indicator of healthy eating habits (10). Nutrition literacy is defined as ‘the degree to which individuals can obtain, process, and understand nutrition information and skills needed to make appropriate nutrition decisions (11). Sufficient studies have examined the relationship between adults’ dietary intake and nutrition knowledge. A positive correlation was observed between nutrition knowledge and a greater intake of fruits and vegetables (12). Other studies observed higher wholegrain intake (13), and a lower intake of sugar-sweetened beverages (14) among individuals exhibiting an adequate nutrition literacy score. Additionally, nutrition knowledge was not only associated with dietary intake but also negatively associated with obesity (15). In this sense, adolescents with adequate nutrition knowledge are provided with the necessary skills to navigate toward healthier food choices and lower rates of obesity and obesity-related diseases.

Several approaches were proposed to determine nutrition literacy or knowledge among adolescents and adults. Unfortunately, the literature did not provide a standard method or tool to assess nutrition literacy. One study suggested that understanding Nutrition Facts labels is an indicator of food choice behavior (16). Others proposed using a nutrition literacy assessment tool that assesses knowledge toward dietary recommendations, nutrient sources of foods, healthy food choices, and diet-disease relationships, which may lead to the overall assessment of adolescent nutritional knowledge (17). The Nutrition Literacy Assessment Instrument (NLit) was developed to assess literacy and numeracy within nutrition contexts and the capability to apply nutrition knowledge and skills (18). Gibbs et al.’s NLit tool assessed five aspects of nutrition knowledge and skills, including Nutrition & Health, Macronutrients (food sources), Household Food Measurement (portion sizes), Food Label and Numeracy, and Food Groups (18). The NLit was validated to assess nutrition literacy among adults with chronic diseases (19). Furthermore, the NLit was used to determine the association between parents’ nutrition literacy and children’s diet quality (8).

Currently, the State of Kuwait is exposed to a remarkable shift in population food intake, characterized by an increase in fast-food outlets. However, this small country is experiencing an increase in adolescent obesity, whereas the estimated prevalence of overweight and obesity among adolescents reached 52.1% (20). In addition, local studies showed that 46.4% of obese adolescents were prediabetics (21). This continued rise in the prevalence of adolescent obesity in Kuwait may lead to an overwhelming increase in the prevalence of obesity-related diseases among adolescents and adults. Although adolescents’ nutrition literacy and knowledge were recognized for their role in dietary habits and adverse health outcomes (22), there have been no efforts to assess nutrition literacy among adolescents in Kuwait.

Given the current prevalence of overweight and obesity among adolescents in Kuwait and the previously demonstrated relationship between nutrition literacy and health outcomes. The primary aim of this study is to assess nutrition literacy among adolescents in Kuwait. Second, this study’s secondary aim is to investigate the effect of adolescents’ nutrition literacy on obesity and dietary behavior.

## 2 Method

### 2.1 Study design and sample

This school-based cross-sectional study recruited 375 participants. The sample included adolescent males and females aged 11–17 years enrolled in public high schools from all six governorates in Kuwait. The minimum required sample size (*n* = 350) was determined so that the sample proportion would be within ±0.05 of the population proportion, with a 95% confidence level.

Our total sample included 210 public high schools distributed among six governorates, and the number of schools varied in each governorate, from 14 to 29 schools. The study participants were selected by two stages: random and convenience sampling. First, random school selection: based on the nature of Kuwait’s public schools, schools in each governorate were stratified by gender (boys’ schools and girls’ schools). We used the Microsoft Excel Random Number Generator to randomly select two schools per gender. Our final school sample included 24 schools. Second, participants’ selection: this stage aimed at selecting students from each school level. Kuwait high schools consist of 3 years of study. A convenience sampling procedure was used to select the students from each year.

Ethical approvals were obtained from the Ethics Committee at the Health Sciences Center, Kuwait University (VDR/EC- 394). Written informed consent was obtained from the parents, and verbal assent was obtained from the adolescents.

### 2.2 Anthropometric measurements

Anthropometric measurements included body weight, height, and waist circumference, which were performed by trained researchers. Body weight was measured to the nearest 100 grams using calibrated portable scales and wearing light clothes. Height was measured in a full standing position, without shoes, to the nearest centimeter using a measuring rope. Waist circumference was measured using a measuring rope, based on the WHO procedure, midway between the lowest ribs and the iliac crest (23).

### 2.3 Nutrition literacy assessment

We used the Nutrition Literacy Assessment Instrument (NLit), which was developed to assess nutrition knowledge and skills in making food choices (18). The instrument included 40 items, categorized into five subscales. (1) Nutrition & Health: assess the ability to associate nutrition intake with health outcomes, scale from 0 to 6 points. (2) Macronutrients; assess knowledge of food energy scale from 0 to 6 points. (3) Household Food Measurement: assess the ability to identify recommended food portions; scale from 0 to 6 points. (4) Food Label and Numeracy: assesses the ability to understand and apply food label information, scale from 0 to 6 points. (5) Food Groups: assess the ability to categorize food items into their nutrition category; scale from 0 to 16 points. Each subscale is scored 0 points for an incorrect answer and 1 point for a correct answer. The total nutrition literacy score is the accumulation of all five subscale scores, ranging from 0 to 40.

### 2.4 Dietary assessment

We determined dietary intake using a single 24-dietary recall. We utilized the Myfood24-Middle East version that uses instructions from food portion photographs and common household measures to estimate food weight (24). Study participants were interviewed by trained researchers to obtain information on food consumed on the previous day. The researcher followed a standard protocol for obtaining dietary information and food item entry within Myfood24. The dietary output sheet included the macro- and micro-nutrient content of each consumed item per participant.

We disaggregated food items into five food groups (fruits and vegetables, meat and poultry, dairy products, and total cereal). Foods and drinks were classified into their groups using the USDA food group classification (25). The disaggregation file classified food as 1 portion = 100 grams. For example, 100 grams of apple = 1 portion of fruit. Therefore, an apple was represented as 1 portion of the fruit group and 0 portions in other food groups. Composite dishes were disaggregated into their constituent parts. For example, a meat wrap was disaggregated into vegetables, meat, and cereal groups based on a standard recipe. Moreover, we classified beverages containing more than 20 g of added sugar as sugar-sweetened beverages.

### 2.5 Covariates

Our primary outcomes were overall nutrition literacy scores and nutrition literacy subscales scores, determined by the NLit tool. Adolescents were divided into three categories in each subscale: 0 to 1 point suggesting a high likelihood of inadequate nutrition literacy, 2 to 3 points suggesting marginal nutrition literacy, and 4 to 6 points suggesting adequate nutrition literacy (18). In the food group subscale, participants were categorized as follows: 0 to 5 points suggest a high likelihood of inadequate nutrition literacy, 6 to 10 points suggest marginal nutrition literacy, and 11 to 16 points suggest adequate nutrition literacy. The literature did not provide a cut-off value for the total nutrition literacy score. Therefore, for this study, we used the median to categorize the study participants as having poor and adequate nutrition literacy. The score median was 29 points, and obtaining a score equal to or above 29 points was considered adequate nutrition literacy. The number of food groups consumed was used as a continuous variable. Sugar-sweetened beverages were analyzed as a continuous variable, representing the aggregation of consumed beverages in milliliters.

We also collected height and weight to measure participants’ body mass index (BMI). BMI was classified into four categories (underweight <5th percentile, normal 5th–85th percentile, overweight or obese 85th percentile, and obese ≥95th percentile) based on the 2022 CDC BMI-for-age and sex-specific.

Other categorical variables, such as sex and smoking status, were analyzed as male and female and smoker and non-smoker, respectively. Residence regions were categorized into six groups based on the governorates in the state of Kuwait (Alahmadi, Alasema, Alfarwaniya, Aljahra, Hawali, and Mubarak AlKabeer). Continuous variables, such as age, BMI, WC, food group consumption, and number of sugar-sweetened beverages, were tested for normality and were normally distributed.

### 2.6 Statistical analysis

We used R statistical software version 3.6.1 for all analyses; the statistical significance threshold was set at *p* < 0.05. Our descriptive analysis reported the prevalence of each categorical variable across our sample size, including nationality, governorate, smoking status, BMI, and nutrition literacy subscales. We used the Wilcoxon test to explore the difference in the overall nutrition literacy score and subscales across the sexes.

To assess the determinants of adolescents’ nutrition literacy, we built a binary logistic regression model using the backward stepwise method. The total NLit score was the dependent variable, which was categorized into adequate and inadequate NLit based on the median score of 29 points, i.e., participants with NLit scores equal to or above 29 points were categorized as an adequate nutrition literacy group.

To assess the effect of nutrition literacy on obesity and dietary choices, we built a linear regression model. We built eight models, one for each dependent variable. Our dependent variables were WC and BMI as measures of obesity. Moreover, food groups (dairy products, meat and poultry, total cereals, fruits, and vegetables), total calorie intake, fiber intake, and sugar-sweetened beverage intake were indicators for dietary choices. Our main independent variable was the NLit score, which was an indicator of nutrition literacy. All eight models were adjusted for sex, place of residence, smoking status, and dietary needs.

## 3 Results

### 3.1 Study sample characteristics

Descriptive statistics of the 375 adolescents are shown in Table 1. The sample included 53.6% females, with a mean population age of 15.6 years. The majority of the study participants were Kuwaiti nationality (94.1%), and a high prevalence were living in the AlAhmadi governorate. The BMI prevalence was 47.5% normal, and 44.5% were overweight and obese. The total nutrition literacy score median is 29 points.

**Table 1:** Study sample characteristics.

Characteristics	All (375) *n* (%)	Female (*n* = 199) *n* (%)	Male (*n* = 176) *n* (%)
Nationality			
Kuwaitis	353 (94.1%)	187 (94.0%)	166 (94.3%)
Non-Kuwaitis	22 (5.9%)	12 (6.0%)	10 (5.7%)
Governorate			
Alahmadi	91 (24.3%)	56 (28.1%)	35 (19.9%)
Alasema	53 (14.1%)	35 (17.6%)	18 (10.2%)
Alfarwaniya	72 (19.2%)	29 (14.6%)	43 (24.4%)
Aljahra	74 (19.7%)	35 (17.6%)	39 (22.2%)
Hawali	46 (12.3%)	31 (15.6%)	15 (8.5%)
Mubarak AlKabeer	39 (10.4%)	13 (6.5%)	26 (14.8%)
Smokers			
No	317 (84.5%)	196 (98.5%)	121 (68.8%)
Yes	58 (15.4%)	3 (1.5%)	55 (31.2%)
BMI categories			
Underweight	30 (8.0%)	15 (7.5%)	15 (8.5%)
Normal	178 (47.5%)	90 (45.2%)	88 (50.0%)
Overweight and obese	167 (44.5%)	94 (47.2%)	73 (41.5%)
The total nutrition literacy score is ^a^	Total median (IQR)	Female median (IQR)	Male median (IQR)
	29 (6)	29 (5.5)	28 (6)

a Accumulation of the five-nutrition literacy assessment tool subscales (8).

Adolescents’ descriptive nutrition literacy scores are shown in Table 2. The majority of adolescents were categorized with adequate nutrition literacy across three sections: nutrition and health, macronutrients, and food groups, 83.3, 76.8, and 89.9%, respectively. Half the study population was categorized with marginal nutrition literacy in the food labels section, 53.3%. Participants were evenly spread between adequate and marginal household measurement scores of 44.0 and 42.7%, respectively.

**Table 2:** The status of adolescents’ nutrition literacy, overall and across sexes.

Nutrition literacy variable	Score classification ^a^	All *n* (%)	Female *n* (%)	Male *n* (%)
Nutrition health score				
	Inadequate	4 (1.1)	1 (0.5)	3 (1.7)
	Marginal	51 (13.6)	25 (12.6)	26 (14.8)
	Adequate	320 (85.3)	173 (86.9)	147 (83.5)
Macronutrient score				
	Inadequate	13 (3.5)	6 (3.0)	7 (4)
	Marginal	74 (19.7)	37 (18.6)	37 (21)
	Adequate	288 (76.8)	156 (78.4)	132 (75)
Household measurement score				
	Inadequate	50 (13.3)	25 (12.6)	25 (14.2)
	Marginal	160 (42.7)	84 (42.2)	76 (43.2)
	Adequate	165 (44.0)	90 (45.2)	75 (42.6)
Food label score				
	Inadequate	100 (26.7)	53 (26.6)	47 (26.7)
	Marginal	200 (53.3)	105 (52.8)	95 (54.0)
	Adequate	75 (20.0)	41 (20.6)	34 (19.3)
Food *g*roups score				
	inadequate	13 (3.5)	3 (1.5)	10 (5.7)
	Marginal	25 (6.7)	13 (6.5)	12 (6.8)
	Adequate	337 (89.9)	183 (92.0)	154 (87.5)

a Nutrition literacy tool subscales score categories (8).

### 3.2 Determine the difference in nutrition literacy categories across sexes

**Figure 1:**
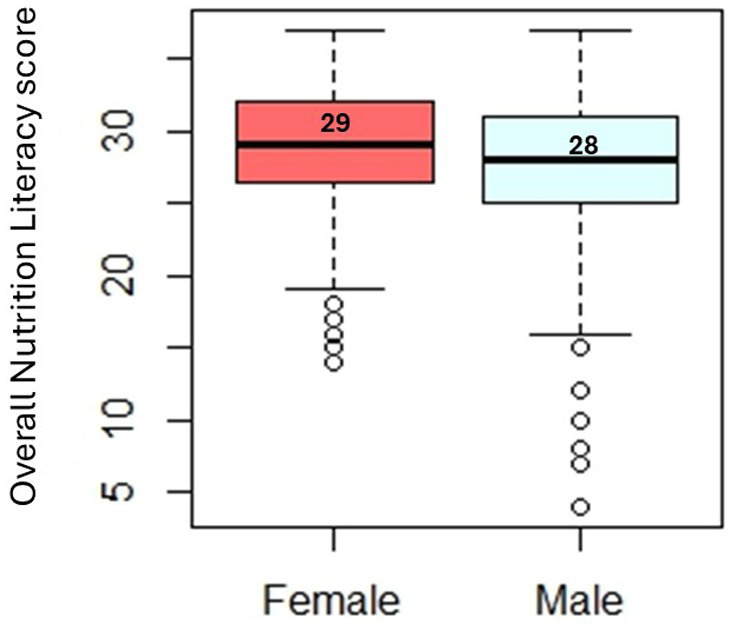
Median comparison of overall nutrition literacy score across sexes, *p*-value = 0.13.

Our findings did not observe a significant difference in overall nutrition literacy scores across sexes (Figure 1). Female adolescents obtained a higher overall median score than males. However, this difference was non-significant.

**Figure 2:**
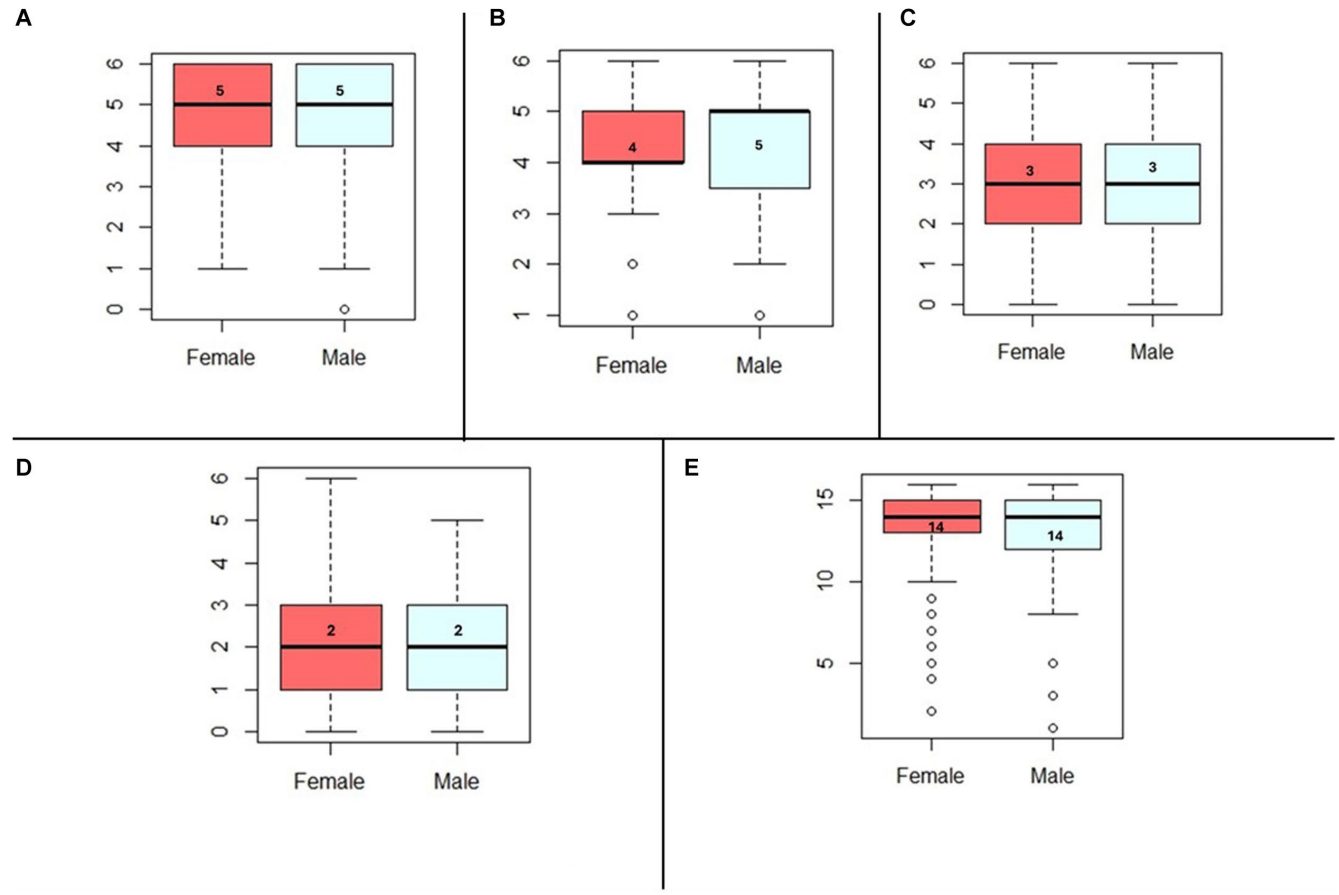
**(A)** Median comparison of nutrition health score across sexes, *p*-value = 0.20. **(B)** Median comparison of macronutrient score across sexes, *p*-value = 0.84. **(C)** Median comparison of Household Measures score across sexes, *p*-value = 0.42. **(D)** Median comparison of Food label score across sexes, *p*-value = 0.67. **(E)** Median comparison of Food Groups score across sexes, *p*-value = 0.20.

Our analysis aimed to determine the difference in nutrition literacy score subscales across sexes (Figure 2). Both groups’ median scores were even in the following subsections: nutrition and health, household measurements, food labels, and food groups. Male macronutrient literacy scores were slightly higher than females’; however, this difference was non-significant.

### 3.3 Assessing the determinants of adolescents’ nutrition literacy

The relationships between study variables and adolescents’ NLit scores are illustrated in Table 3. Adolescents were categorized into adequate and inadequate NLit scores based on the score median of 29 points. No association was observed between adolescent gender and NLit scores. No association was observed among residential areas except in AlJahra (OR: 0.2 *p* < 0.05). Poor NLit scores were obtained among adolescents living in AlJahra. Smokers were more likely to obtain a lower NLit scores score (OR: 0.4, *p* < 0.05). Moreover, adolescents who reported following special diet plans or requirements were likelier to obtain an adequate NLit score (OR: 5.0, *p* < 0.05). No association was observed between WC and NLit scores. However, the association between BMI and nutrition literacy was slightly significant (OR: 0.9, *p*-value: 0.07). This might suggest that a higher BMI may lead to obtaining a lower nutrition literacy score.

**Table 3:** The determinants of adolescents’ nutrition literacy.

	OR ^a^ (95% CI)	*p*-value ^b^
Sex (Reference female)		
Male	1.01 (0.02, 50.0)	0.96
Place of residence (Reference Alahmadi)		
Alasema	1.6 (0.78, 3.4)	0.16
Alfarwaniya	0.8 (0.41, 1.6)	0.56
Aljahra	0.2 (0.11, 0.5)	0.0003 *
Hawali	1.0 (0.50, 2.3)	0.81
Mubarak AlKabeer	1.4 (0.61, 3.3)	0.37
Smoking (Reference: non-smoker)		
Smoker	0.4 (0.23, 1.0)	0.05 *
Following a weight loss or special dietary needs (Reference: no)		
Yes	5.0 (2.0, 12.9)	0.0004 *
Waist circumference (cm)	1.0 (0.98, 1.0)	0.29
Body Mass Index (Kg/m^2^)	0.9 (0.88, 1.0)	0.07

a Adjusted odds ratio.

b Variables entered: sex; place of residence; body mass index; waist circumference; smoking status; dietary needs.

*Significant at *p*-value < 0.05.

### 3.4 The association between nutrition literacy and food group intake

The effect of nutrition literacy on obesity and dietary choices is illustrated in Table 4. Each reported coefficient and CI in Table 4 represent the results of an individually built linear regression model. The nutrition literacy score was not associated with obesity which was measured by BMI and WC. No association was observed between nutrition literacy score and dietary choices, measured by food group consumption, total calorie intake, and fiber intake. The nutrition literacy score was negatively associated with the consumption of sugar-sweetened beverages. Suggesting that adolescents with adequate nutrition literacy scores (> 29 points) are less likely to consume sugary beverages.

**Table 4:** Assessing the effect of nutrition literacy score on body mass index, waist circumference, and food groups ^a^.

	*β* ^b^^,^^c^	95% CI
Anthropometrical measurement		
Body mass index (kg/m^2^)	−0.04	−0.19, 0.11
Waist circumference (cm)	0.04	−0.33, 0.4
Food Group ^d^		
Fruits and vegetables	0.007	−0.01, 0.02
Dairy products	−0.01	−0.04, 0.009
Meat, poultry, and fish	0.01	−0.02, 0.05
Total calorie intake (Kcal)	0.75	−16.22, 17.73
Fiber (grams/ 1,000 kcal)	−0.02	−0.07, 0.03
Total sugar-sweetened beverages ^e^	−6.35*	−10.9, −1.71

* *p* < 0.001.

a Model dependent variable (BMI, WC, or each food group).

b Reported coefficient and CI for the main independent variable: total nutrition literacy score.

c Model adjusted for sex; place of residence; smoking status; dietary needs.

d Food groups classified based on the USDA (25).

e Amount of sugar-sweetened beverage in milliliters.

## 4 Discussion

This school-based cross-sectional study assessed the adolescents’ nutrition literacy in Kuwait and investigated the NLit score association with demographic factors and dietary intake. To the best of the authors’ knowledge, this is the first study to assess nutrition knowledge among this age group in Kuwait. This is significantly important given the continuous rise in adolescent obesity (20) and obesity-related diseases (21) in Kuwait. In addition to the previously observed association between nutrition literacy and obesity (26, 27). This study enrolled a convenience sample of 375 adolescents with a mean age of 15.6 years, recruited from all six governorates in the State of Kuwait. Study findings showed that the majority of adolescents were categorized as having adequate nutrition literacy in four nutrition literacy subscales and marginal nutrition literacy in the food label and numeracy subscale. No difference was observed between male and female adolescents in terms of total nutrition literacy score and across subscales. Moreover, nutrition literacy score was associated with adolescents’ place of residence, smoking status, and having special dietary needs. A slightly significant association was observed between NLit score and BMI. Nutrition literacy score was negatively associated with the consumption of sugar-sweetened beverages, and adolescents with an adequate NLit score were less likely to consume sugar-sweetened beverages. No association was observed between nutrition literacy and consumption by other food groups.

The availability of multiple nutrition literacy assessment tools makes it a challenge to compare our study results with national or regional studies (28). In the current study, adolescents had a median NLit score of 29 out of 40 (72.5%). These scores were comparable to the ones reported in studies previously undertaken in the UK (72.7%) (17). This study score is higher than that of regional studies, where 44.6% of Saudi (29) and 62% of Iranian (30) adolescents had inadequate nutrition literacy. This variation in the level of nutrition literacy might be associated with the differences in nutrition literacy assessment tools. For example, the UK-based study (17) utilized the General Nutrition Knowledge Questionnaire (GNKQ-R). The GNKQ-R tool is subdivided into four subscales comparable with our study’s nutrition literacy assessment tool. These subscales are food and nutrition sources and the relationship between nutrition and diseases. On the other hand, the Saudi-based study (29), assessed nutrition literacy using the Adolescent Nutrition Literacy Scale (ANLS). The ANLS assessed three components: functional, interaction, and critical nutrition literacy.

This present study utilized the NLit tool, which is designed to capture five elements associated with nutrition knowledge: Nutrition & Health, Macronutrients, Household Food Measurement, Food Label & Numeracy, and Food Group (18). A closer investigation of those subscales showed that adolescents in Kuwait are classified with adequate nutrition literacy across three sections: nutrition and health, macronutrients, and food groups, 83.3, 76.8, and 89.9%, respectively. However, 53.3 and 42.7% of adolescents in Kuwait were classified as having marginal nutrition literacy in the food labels and household measurement (portion sizes) subscales, respectively. These findings may suggest that adolescents in Kuwait clearly understand the association between nutrition and health outcomes, in addition to understanding the role and sources of macronutrients. This study’s findings were comparable with those of the UK adolescent study, where the study participants had adequate knowledge of diet-disease relationships (17). Moreover, this study’s findings were consistent with a United States adolescent study, where 59% of girls and 52% of boys correctly understood food label facts (16).

Place of residence, smoking, and dieting were significant predictors of the NLit score in the present study sample. This is consistent with previous evidence showing that family socio-demographic factors are significant predictors of children’s nutrition literacy (29, 31). Researchers suggested that education inequality among different residential areas could influence adolescents’ nutrition literacy (29). However, education inequality might not be a possible explanation for the inadequate NLit score we observed in the Aljahra governorate. This is because we recruited adolescents from public schools, receiving a standardized education level. Therefore, education inequality might not be a contributing factor. This variation in NLit score could be explained by independent school activities and efforts to improve nutrition education, such as guest nutrition education lectures, health field trips, and sports days (32, 33). In addition, parents, especially mothers, are important influencers in shaping early childhood eating behavior, and nutritional knowledge, which might be tracked into adolescent phase (34). Another study suggested family income, parents’ education, and the number of family members are significant predictors of nutrition literacy (35). Therefore, further investigations are required to explain our observations on the NLit score’s association with place of residence.

Our analysis observed a slightly significant association between BMI and NLit score, suggesting that Kuwaiti adolescents’ BMI might predict their nutrition literacy score. Our study’s findings were consistent with previous studies where obesity was a significant predictor for inadequate nutrition literacy (26). Various factors could explain this variation in the influence of obesity on nutrition literacy. First, variation in nutrition literacy assessment tools might affect the study results. Second, the confounding effect of other factors should be considered, such as parental nutrition literacy (8) and home and school environment (36, 37).

Although we observed adequate nutrition knowledge among our study sample, the main question is whether nutrition knowledge influences adolescent dietary choices. A systematic review showed a positive association between adolescent nutrition knowledge and fruit and vegetable intake (38). Other studies observed a significant association between meat intake and nutrition literacy (32). Wojcicki and Heyman stated that 92.4% of adolescents were aware of the Food Guide Pyramid. However, less than 25% chose food items based on reading nutrition facts labels (39). Another study suggested that providing caloric and fat content information to fast food items did not modify adolescents’ fast food ordering behavior (40). This variation in study results could be explained by differences in dietary assessment methods, diet reporting bias, and variations in nutrition education across study participants. Our findings are consistent with previous studies, which showed that adequate nutrition literacy (NLit >29 points) was not associated with food choices, measured by food group consumption, fiber intake, and total caloric intake. Therefore, our findings and previous findings may suggest that adequate nutrition literacy among adolescents in Kuwait might not affect their food choice behavior.

Our findings observed a significant negative association between sugar-sweetened beverage intake and nutrition literacy score. This suggests that adolescents with an adequate NLit score exhibited a lower consumption of sugar-sweetened beverages, such as non-diet fizzy drinks, sugar-sweetened juices, and energy drinks. These findings were consistent with a randomized trial among adolescents, showing that nutrition education interventions significantly reduced consumption of sugary beverages (41).

Our study showed that 44.5% of Kuwaiti adolescents were either overweight or obese, which is consistent with previous local investigations (20). In addition, our study showed that nutrition literacy is not a predictor of this prevalence of overweight and obesity. Previous studies also did not observe an association between BMI and nutrition literacy among adolescents. However, this association was significant among male adolescents, only (27). The non-significant association between obesity indicators and nutrition literacy could be explained by several factors. First, this study’s findings and previous observations suggested that nutrition literacy might not influence dietary choices (39, 40). This might suggest that adequate nutrition knowledge might not guide adolescents to healthier food choices that reduce their risk of obesity. Second, nutrition literacy was not associated with fiber or total caloric intake among adolescents in Kuwait. However, both fiber and reduced caloric intake have been identified for their roles in preventing obesity (42). Consequently, if adequate nutrition knowledge does not affect fiber and caloric intake, it may not have a direct impact on reducing obesity risk.

Our study findings had several implications. First, we provided evidence that the importance of nutrition literacy among adolescents lies in its potential to promote healthy eating habits and prevent obesity. Similarly, enhancing nutritional knowledge improves adolescents’ ability to make informed food choice decisions, which may reduce the burden of obesity and obesity-related diseases. Second, assessing nutrition literacy and the underlying associated factors is crucial before initiating health promotion or nutrition education programs. By doing so, we can ensure that schools’ nutrition education and health promotion efforts in Kuwait are built on scientific evidence and are not driven by public trends. Finally, our findings suggested that a large percentage of Kuwaiti adolescents demonstrated adequate nutrition literacy. Therefore, future research should investigate why, with adequate nutrition knowledge, adolescents in Kuwait still experience a continuance rise in the prevalence of obesity.

### 4.1 Strength and limitation

To the best of our knowledge, this is the first study to assess nutrition literacy among adolescents in Kuwait and the first to explain the impact of nutrition literacy on obesity and food intake in this population. Therefore, our analysis could serve as a foundation for developing health promotion programs and implementing school-based nutrition education policies.

Our study findings should be interpreted with caution due to several limitations. First, the cross-sectional design does not establish causal relationships between the study variables. Second, factors such as parental nutritional literacy, family income, and existing school nutrition education programs were not assessed, which may have influenced nutrition literacy score scores. Third, reporting bias might affect the analysis of dietary associations. Finally, the nutrition literacy tool was not validated for Arabic-speaking adolescents.

## 5 Conclusion

In conclusion, we found that a large percentage of Kuwaiti adolescents demonstrated adequate nutrition literacy in the majority of aspects of the literacy questionnaire. However, the majority of adolescents demonstrated lower-than-adequate nutrition literacy when it came to understanding food labels. Nutrition literacy scores were influenced by factors such as place of residence, smoking status, and BMI. Additionally, nutrition literacy was negatively associated with the consumption of sugar-sweetened beverages. These findings suggest that enhancing nutrition literacy among adolescents could help reduce the risk of obesity and improve food choices.

Since adolescence is an important phase for establishing dietary patterns and preventing obesity, developing health promotion strategies based on our observation could help improve the overall prevalence of obesity among Kuwaiti adolescents. Therefore, our findings support the initiation of school-based nutrition education programs and the development of nutrition policies.

## Data Availability

The raw data supporting the conclusions of this article will be made available by the authors, without undue reservation.
